# Understanding Pre-Type 1 Diabetes: The Key to Prevention

**DOI:** 10.3389/fendo.2018.00070

**Published:** 2018-03-06

**Authors:** Laura M. Jacobsen, Michael J. Haller, Desmond A. Schatz

**Affiliations:** ^1^Division of Endocrinology, Department of Pediatrics, University of Florida, Gainesville, FL, United States

**Keywords:** prevention, type 1 diabetes, staging, mechanisms, autoimmune diseases

## Abstract

While the incidence of type 1 diabetes continues to rise by 3% each year, the ability to prevent this disease remains elusive. Hybrid closed loop devices, artificial pancreas systems, and continuous glucose monitoring technology have helped to ease the daily burden for many people living with type 1 diabetes. However, the artificial pancreas is not a cure; more research is needed to achieve our ultimate goal of preventing type 1 diabetes. The preceding decades have generated a wealth of information regarding the natural history of pre-type 1 diabetes. Islet autoimmunity in the form of multiple autoantibodies is known to be highly predictive of progression to disease. Staging systems have been devised to better characterize pre-type 1, direct mechanistic understanding of disease, and guide the design of prevention studies. However, there are no evidence-based recommendations for practitioners caring for autoantibody patients other than to encourage enrollment in research studies. Close monitoring of high-risk patients in natural history studies markedly reduces diabetic ketoacidosis rates at diagnosis and research participation is critical to finding a means of preventing type 1 diabetes. The discovery of an effective preventative strategy for type 1 diabetes will justify universal risk screening for all children.

## Introduction

As is the case for type 2 diabetes, the incidence and prevalence of type 1 diabetes is increasing annually. It is estimated that more than 542,000 children worldwide have type 1 diabetes. With the diagnosis of type 1 diabetes rising by 2–3% per year, 86,000 children are expected to developed type 1 diabetes each year (1, 2). Recent data from Thomas et al. suggests that this is an underestimate when both children and adults diagnosed with type 1 diabetes are included. They estimate that over 40% of all new cases of type 1 diabetes occur over the age of 30 years (3). While advancements and innovation are occurring for those affected with type 1 diabetes in the artificial pancreas arena and therapeutic interventions for new-onset diabetes clinical trials, a focus on the prevention of type 1 diabetes is crucial. Prevention revolves around the identification and interdiction of this immune-mediated process.

Intensive insulin therapy in the Diabetes Control and Complications Trial has led to decreases in, but not the absence of, microvascular and macrovascular complications of diabetes (4). Insulin pump therapy and continuous glucose monitoring are setting the course for widespread use of a closed loop system, but insulin itself is not a cure. Despite advances, recent data from the T1D Exchange show no improvement in metabolic control over the past 5 years (5).

The etiology and precise mechanisms leading to type 1 diabetes remain elusive. Nevertheless, considerable progress has been made in our understanding of the natural history of “pre-type 1 diabetes.” Such natural history study advances have led to the earlier diagnosis of type 1 diabetes and less diabetic ketoacidosis (DKA) at onset in those followed prospectively (6–8). These studies are a platform for studying mechanisms and staging of the disease to enable use of preventative therapies. No formal pre-type 1 diabetes evidence-based guidelines exist, but as endocrine and diabetes providers, we are responsible for (1) understanding the risk of progression to type 1 diabetes, (2) preventing DKA, and (3) advocating for prevention trials, mechanistic and natural history studies, and their continued funding and support.

## At-Risk Population

To prevent type 1 diabetes, our understanding of the natural history of pre-type 1 diabetes and the mechanisms culminating in the autoimmune destruction of beta cells must continue to advance. Large international cohorts have been studied from birth in both relatives of patients with type 1 diabetes and more recently in the general population who are at high genetic risk. These studies have selected infants based on high-risk human leukocyte antigen (HLA) alleles, most commonly HLA-DR3/4, DQB1*0201/DQB1*0302. Monozygotic twins have a lifetime 50–70% risk of developing type 1 diabetes (9). In the US, the risk of developing type 1 diabetes is 1 in 20 in first-degree relatives and in the general population is 1 in 300 (9). By studying these individuals over time, risk predictors of progression to type 1 diabetes were determined, and none were more pronounced than the presence of islet autoantibodies. Islet autoantibodies develop in 90–95% of those destined to develop type 1 diabetes (10). These include islet cell autoantibodies (ICA) detected by indirect immunofluorescence (11) and insulin autoantibodies (IAA), glutamic acid decarboxylase autoantibodies (GADA), and insulinoma associated-2 autoantibodies (IA-2A) measured by radiobinding assays, and more recently zinc transporter 8 autoantibodies (10). Newer, more sensitive assay methods including electrochemiluminescence have been developed (12).

The forethought to initiate long-term natural history studies greatly increased our knowledge of islet autoimmunity, the precursor of clinical disease. Large birth cohorts including Germany’s BABYDIAB (started in 1989), Finland’s Diabetes Prediction and Prevention (DIPP; started in 1994), and Colorado’s Diabetes Autoimmunity Study in the Young (DAISY; started in 1993) demonstrated a peak in islet autoimmunity development within the first 2–5 years of life and more rapid disease progression in those who developed autoantibodies in these early years compared to later childhood and adulthood (13, 14). IAA development occurs first in these young children with IgG1 subclass predominance and is more likely to be associated with the DR4 allele (13, 15). While islet autoantibodies detected in cord blood are most likely maternal in origin, Germany’s BABYDIAB demonstrated lower risk in offspring of mothers with type 1 diabetes who had GADA or IA-2A in cord blood than those who were autoantibody negative offspring of type 1 diabetes mothers (16). In the most definitive study to date, The Environmental Determinants of Diabetes in the Young (TEDDY) study (started in 2004) is examining gene/environment interactions and subsequent development of islet autoimmunity and clinical disease (17). To date, this multi-country study (Germany, Finland, Sweden, US), has confirmed what appears to be two waves of separate islet autoantibody appearance—IAA within the first 18–24 months and GADA around age 3 years (15), as well as confirmed the correlation with genetic risk and importantly age (18, 19). Recent publications identify possible risk augmenters seen within the TEDDY cohort including non-HLA genes, single nucleotide polymorphisms, and other autoimmune diseases (20).

**Figure 1:**
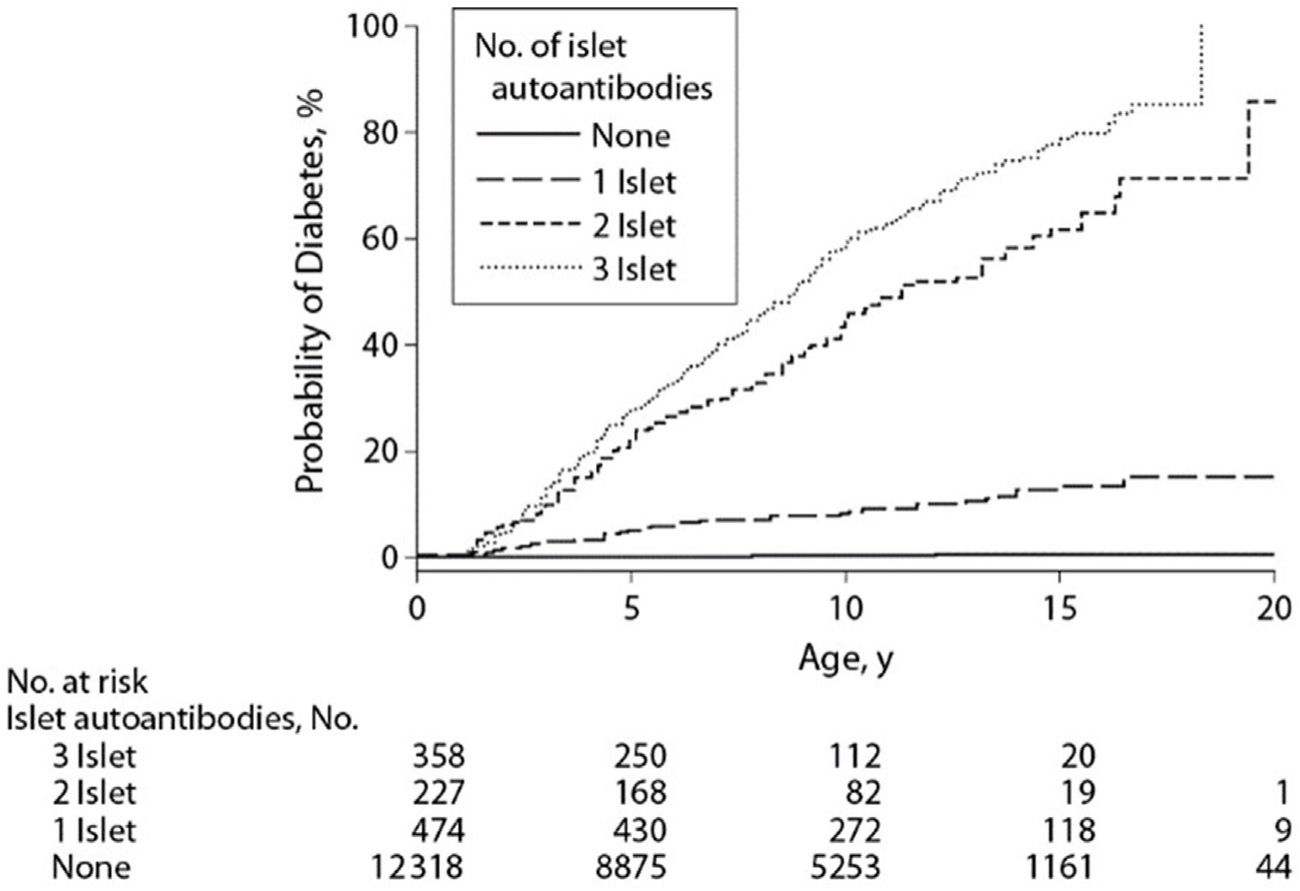
Development of diabetes in children stratified for islet autoantibody outcome. The numbers at risk represent the children receiving follow-up at age 0, 5, 10, 15, and 20 years. Reproduced with permission from Ziegler et al. (21).

Combining data derived from the aforementioned DIPP, DAISY, and, BABYDIAB birth cohort studies, Ziegler et al. demonstrated the risk of progression to type 1 diabetes based on the age of appearance of autoimmunity and number of autoantibodies. This young cohort of genetically high-risk children with one autoantibody had a 10-year risk of progression to diabetes of 14.5%. Children with two or more autoantibodies were at markedly increased risk of progression to type 1 diabetes at 5-year (43.5%), 10-year (69.7%), and 15-year (84.2%) follow-up. Sixty percent of children with multiple autoantibodies progressed to diabetes (median age of 6.1 years) compared to 10% of children with a single autoantibody (median age 5.2 years) (Figure 1) (21).

## Staging of Type 1 Diabetes

**Figure 2:**
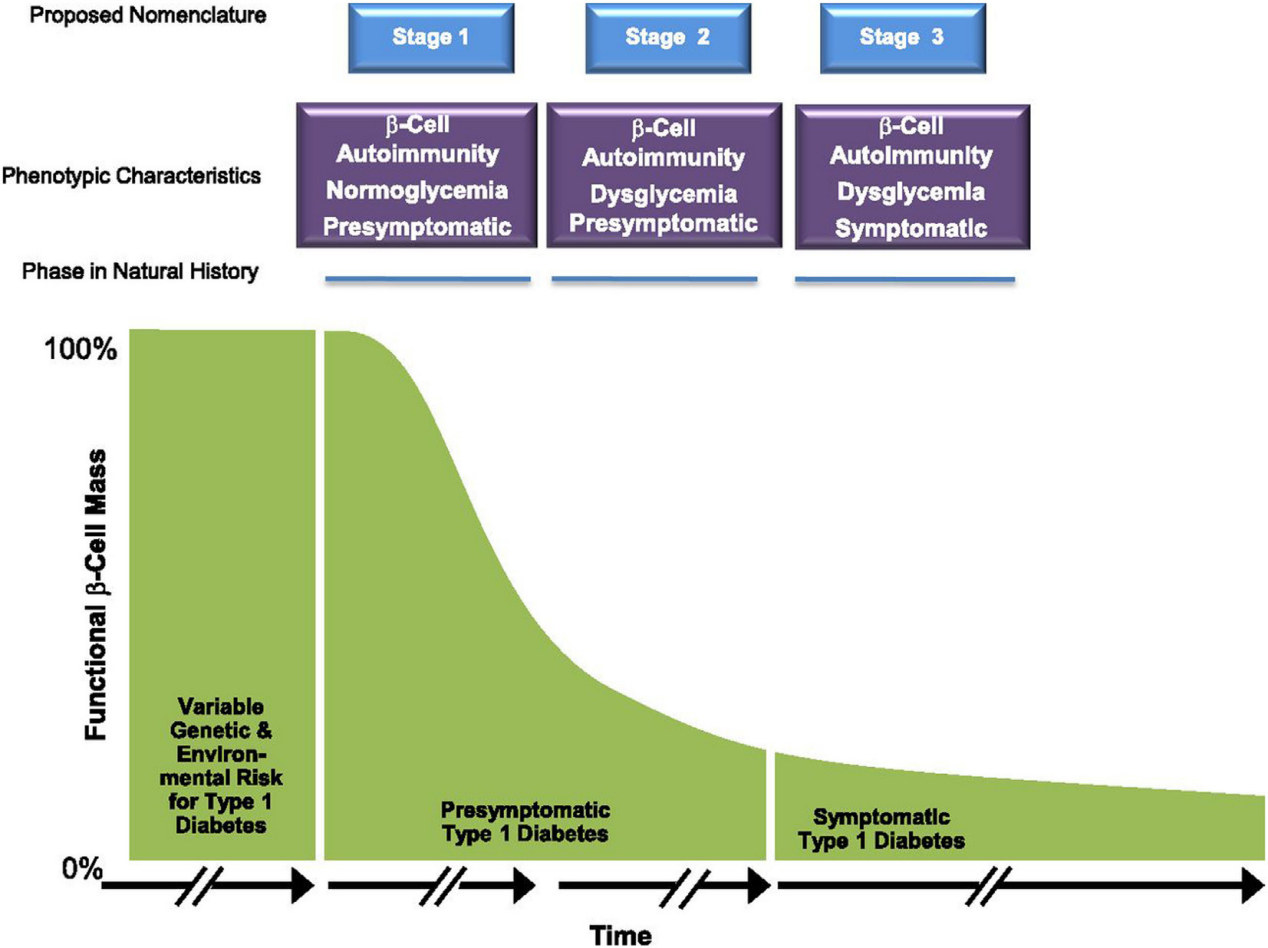
Early stages of type 1 diabetes. Reproduced with permission from Insel et al. (23).

These birth cohort studies, in addition to non-birth cohorts such as the large Diabetes Prevention Trial-Type 1 (DPT-1, 1994–2003) and the Type 1 Diabetes TrialNet Pathway to Prevention (PTP; 2004–present) studies have allowed us to further characterize the time period before diagnosis. Although the actual diagnosis of diabetes has traditionally been based on American Diabetes Association (ADA) criteria (22), it is clear that the onset of the disease *per se*, often occurs years before the onset of symptoms. Thus, pre-type 1 diabetes is a unique physiologic state where autoimmunity is present and progression to metabolic derangement and clinical onset can be predicted especially in younger children and adolescents. As such, the ADA, JDRF, and Endocrine Society released a joint position statement for the staging of pre-type 1 diabetes (Figure 2) (23). Stage 1 is defined by the presence of 2 or more islet autoantibodies with normoglycemia (normal glucose tolerance on 2-h OGTT). Stage 2 shows progression to dysglycemia (impaired glucose tolerance) in the setting of 2 or more islet autoantibodies, and stage 3 occurs when a patient meets ADA criteria for the diagnosis of diabetes.

Why is this distinction important? The overall goal of all those who care for children and adults with type 1 diabetes is to cure the disease (obviously preventing its recurrence) as well as prevention of the disease in those at risk and destined to subsequently develop clinical onset. Participants screened and followed as part of natural history studies whether enrolled in prevention trials or not have a decreased incidence of DKA at diagnosis compared to those screened but not followed and those not screened (6–8). While DKA rates at onset of type 1 diabetes vary widely between countries (16–67%) (24), there has clearly been a decrease shown within the TEDDY study; rates of DKA in those under 5 years of age were significantly less (11.3%) compared to national population-based registries (US SEARCH 36.4%, Swediabkids 16.9%, Finnish Pediatric Diabetes Register 18.7%, and German DPV Register 32.2%) (6). This lower rate of DKA was even more significant in the youngest age group (<2 years old) where DKA is more common and carries a higher mortality (DKA rates of 15% in TEDDY versus 39.5–54% in population-based registries) (6). Thus, as a consequence of close monitoring in natural history studies, there is decreased morbidity and mortality. Several recent studies have also demonstrated long-term glycemic benefits seen for those patients who did not experience the severe metabolic derangement of DKA at diagnosis (25, 26).

Understanding the likelihood of progression to disease in different groups is crucial. In prevention studies, a clinical endpoint, such as type 1 diabetes, can take years to reach and result in lengthy, expensive clinical trials. Limiting enrollees to those already in stage 2 may see endpoints reached more rapidly. That said, curtailing studies to those with advanced autoimmunity limit those available to enroll and can slow recruitment. Additionally, non-interventional, mechanistic-focused studies in stage 1 and 2 subjects can be designed to further our understanding of the etiopathogenesis of disease. The classification of pre-type 1 diabetes provides a uniform way in which researchers and clinicians can converse while promoting more individualized medical management (23). Clinical trial design, subject selection, and risk versus benefit analysis can all be improved by the use of these risk-stratified groups.

Biomarkers, if validated, would be useful in understanding the natural history of the disease, heterogeneity, and as well as in clinical trials to shorten studies. C-peptide preservation is well studied as a metabolic endpoint in intervention trials. Samples collected during OGTT can be used as absolute values (fasting and peak C-peptide) or as part of a multivariable equation such as Index60 derived from a proportional hazards regression model as predictors of progression to type 1 diabetes (27–29). Markers of beta cell-related stress, damage, and death are currently under investigation.

Our knowledge of rates of progression, risk factors and the heterogeneity of the disease has been greatly advanced by these studies. Recently, it has been proposed that stage 1 and 2 could also be referred to by the terminology autoimmune beta cell disorder (ABCD), though this is new and may be controversial nomenclature (30–32). Semantically, ABCD may promote understanding among primary care providers, pharmaceutical companies, and funding organizations as to the importance of this unique physiologic state and increase awareness and urgency to prevent this disease. Additionally, as the population of islet autoantibody-positive patients grows, what will be the appropriate counseling, monitoring, and management of these patients?

The cost of population screening and the parental anxiety associated with early monitoring for a disease must also be kept in mind (33). Parental anxiety studied within TEDDY has shown increased anxiety with genetic screening that decreases if no further risk is incurred (islet autoimmunity); however, with increasing positive islet autoantibodies increases in parental anxiety may occur (and slowly lessens with time) (34). Early population-based genetic and/or autoantibody screening programs are currently in progress (35). Public awareness campaigns for the earlier diagnosis of type 1 diabetes have shown mixed results but with some decreasing DKA at onset of type 1 diabetes (36).

## Practical Approach to Pre-Type 1 Diabetes

A practical approach to the management of islet autoantibody positivity starts with how islet autoantibody positivity is determined. This may occur through (1) testing of islet autoantibodies in patients found to have a mildly elevated blood glucose found incidentally (not meeting criteria for diabetes), (2) hyperglycemia detected during an acute illness that resolves but in whom autoantibody testing was done, (3) screening in the setting of multiple other autoimmune conditions, (4) screening of family members of probands diagnosed with clinical type 1 diabetes as part of a research study, or (5) population screening (only in the context of research). After confirmation of either one or more islet autoantibodies (specific autoantibody assays mentioned previously), the patient should be counseled if possible (based on age, number/type of autoantibodies and glycemia status) and referred to centers participating in available research studies (in the US, the NIH-funded TrialNet umbrella). If the patient does not qualify for any trials or does not wish to participate in a trial or research follow-up, the primary care provider, endocrinologist, or diabetologist should consider performing a hemoglobin A1c (HbA1c) and/or random/fasting/post-prandial blood glucose self-monitoring. An OGTT may be done to detect early clinical diabetes (Stage 3).

Initiation of insulin is not recommended in the pre-type 1 phase. Initiation of insulin early in disease was previously thought to provide for beta cell rest and recovery even without severe metabolic derangement or markedly elevated HbA1c. More recent studies, such as the DPT-1, have shown that this is not the case, and others looking at intensive insulin therapy initiated shortly after diabetes diagnosis fail to show increased preservation of C-peptide compared to conventional treatment (37, 38). Management questions that will require more study include the optimal time to start insulin in a patient diagnosed with stage 3 disease without symptoms (“silent diabetes”). Other important areas of pre-type 1 diabetes management to consider include the potential benefit of intense diet and exercise or GRAS (Generally Regarded as Safe) therapies. Will there be a role for adjunctive therapies other than insulin such as glucagon-like peptide-1 agonists or metformin? These and other preventative therapies are also being studied.

## Prevention Trials in Type 1 Diabetes

### Recently Completed Studies

Prevention trials may have multiple endpoints and the populations treated may differ. Those found to be genetically at risk for developing islet autoimmunity are targeted with primary prevention strategies that are typically of low risk. Secondary prevention studies aim to slow down or halt the destruction of beta cells in those who have islet autoantibodies. Multiple approaches have been used with limited success to date (Table 1). These include dietary changes, antigen-based therapy, immunomodulatory, and immunosuppression therapies. Primary dietary prevention strategies beginning in the mid-1990s included the Trial to Reduce IDDM in the Genetically at Risk evaluating the role of a hydrolyzed casein-based formula (free of intact bovine insulin) compared to cow’s milk-based formula, BABYDIET looking at a gluten-free diet in the first year of life, the Finnish Dietary Intervention Trial for the Prevention of Type 1 Diabetes (FINDIA) with insulin-free bovine formula, and the TrialNet Nutritional Intervention to Prevent Type 1 diabetes study with docosahexaenoid acid. All studies failed to show efficacy other than FINDIA which demonstrated some delay in the development of autoantibodies (39–41).

**Table 1:** Overview of recently completed, current and planned clinical trials aimed at prevention of type 1 diabetes.

Recently completed	Trial to Reduce IDDM in the Genetically at Risk (TRIGR)
	BABYDIET study in Germany
	Finnish Dietary Intervention Trial for the Prevention of Type 1 Diabetes (FINDIA)
	TrialNet Nutritional Intervention to Prevent (NIP) Type 1 diabetes study
	Type 1 Diabetes Prediction and Prevention (DIPP) study in Finland
	Pre-POINT (Primary Oral/Intranasal INsulin Trial) and Pre-POINT-early in Germany
	Diabetes Prevention Trial-Type 1 (DPT-1)
	TrialNet Oral Insulin study
	Australian Intranasal Insulin Trial-I (INIT I)
	Diabetes Prevention—Immune Tolerance (DIAPREV-IT) study
	European Nicotinamide Diabetes Intervention Trial

Current	TrialNet Teplizumab (anti-CD3) trial
	TrialNet Abatacept (CTLA4-Ig) trial
	The CoRD Study with autologous cord blood in Australia
	DIAPREV-IT2 study
	Australian Intranasal Insulin Trial-II (INIT II)

Future	TrialNet Aldomet (methyldopa) study
	TrialNet Hydroxychloriquine
	TrialNet Rituximab and Abatacept
	Fr1da Insulin Intervention in Germany
	Adjunctive therapies such as glucagon-like peptide-1 receptor agonists

Antigen therapy was established with the hope of inducing peripheral tolerance by exposure of the naïve immune system to an antigen found in the target organ (beta cell) or through induction of anergy of already present autoreactive T cells (42, 43). The Type 1 Diabetes Prediction and Prevention (DIPP) study in Finland screened cord blood samples for high-risk HLA genotypes and followed children for the subsequent development of autoantibodies. Children were treated with intranasal insulin or placebo and the outcome was no different between groups. In Germany, the Pre-POINT (Primary Oral/Intranasal INsulin Trial) study administered different doses of oral insulin (and intranasal insulin) to high-risk HLA individuals prior to the development of autoantibodies. This small study demonstrated mechanistic/immunological effects including elevated serum IgG and salivary IgA binding to insulin and an increase in regulatory T cells (39). This led to the ongoing Pre-POINT-early study including children 6-months to 2-years old looking for induction of CD4+ T cell and antibody responses against insulin with dose escalation of oral insulin.

Insulin was first targeted as an autoantigen in the late 1980s starting in the non-obese diabetic (NOD) mouse model. In the early 1990s, two insulin-based therapies were conducted in the DPT-1 network. In separate studies, oral insulin and intravenous/subcutaneous insulin were administered to those of intermediate and high-risk, respectively. No difference was seen except in an *ad hoc* analysis of a subgroup—those with positive ICA, elevated IAA titers (≥80 nU/mL), and normal glucose tolerance—a projected delay of 4.8 years in onset was observed. An even greater delay was observed in those with higher IAA levels (>300 nU/mL). The protective effect continued even after the end of the study (39). The large TrialNet Oral Insulin study (2007–2016) recently completed and demonstrated no effect in the overall cohort although a delay was noted in a stratum of high-risk subjects (with loss of first phase insulin response) treated with oral insulin (44). Further analyses through a small mechanistic trial of participants receiving oral insulin has completed and is being analyzed. Other modes of insulin delivery aiming to achieve immune tolerance have also been employed including intranasal insulin administration in the Australian Intranasal Insulin Trial-I and II (INIT I and II) (45).

Glutamic acid decarboxylase (GAD), another islet autoantigen, as a vaccination in the new onset and prevention time period has failed to provide preservation of beta cell function and effective delay in type 1 diabetes onset, respectively (39). The use of GAD together with an aluminum adjuvant (Diamyd^®^), in the Diabetes Prevention—Immune Tolerance (DIAPREV-IT) study, has shown increases in GADA titers but no delayed onset of disease (46). The addition of high dose vitamin D to Diamyd for the DIAPREV-IT2 study is ongoing. A non-antigen-based therapy, the European Nicotinamide Diabetes Intervention Trial study, in which relatives who had developed islet cell antibodies were randomized to 5 years of nicotinamide versus placebo, showed no difference in the rate of diabetes development (39).

### Current Studies

In addition to the oral insulin and GAD-alum studies mentioned above there are other ongoing antigen-based prevention (and intervention) trials including those using multiple peptide mixtures from known islet autoantigens with the aim of inducing immunological tolerance to beta cells (47). More recently, there has been a focus on immunologic modulation in prevention studies after promising efficacy results in new-onset studies (38). Attempts to restore self-tolerance, promote Tregs, and reduce Teff have been evaluated with several different classes of drugs including anti-CD3. Based on data from well-designed new-onset studies (Protégé and AbATE trials) (48), TrialNet has just completed enrollment in a high-risk population of relatives with two or more autoantibodies and dysglycemia (stage 2 disease) using anti-CD3 (tepiluzimab). Abatacept, CTLA4, co-stimulation blockade was chosen for transition from intervention to prevention trials after it demonstrated a slowed rate of beta cell decline that was maintained 1 year after therapy cessation (39). The TrialNet Abatacept prevention study is underway and is still recruiting individuals with stage 1 disease (multiple autoantibodies and normal glucose tolerance).

A cellular therapy approach seeking the promotion of tolerizing Treg cells using autologous cord blood is underway in Australia (The CoRD Study). This open label pilot study is recruiting multiple islet autoantibody-positive first-degree relatives. Simultaneously, the DIAPREVI-IT2 and the INIT II studies mentioned above have built off their predecessors and are looking to add new therapies or enlarge the group studied.

### Future Studies

Once efficacy, safety, and feasibility (and hopefully mechanism) is demonstrated in new-onset type 1 diabetes patients receiving immune and other therapies they should be moved into the prevention arena. Due to the number of potential therapeutic targets—both immune and non-immune—multi-agent (cocktail) therapy targeting multiple aspects of this disease is likely to be needed. The timing of initiation and duration of treatment are also important areas of study.

Type 1 Diabetes TrialNet has recently expanded to other therapeutics that have been approved and tested safe in other conditions and populations. One is the use of methyldopa to inhibit the communication between antigen presenting cells through MHC Class II signaling in susceptible HLA-DQ8 haplotypes. This focused, small mechanistic study will enroll participants with HLA-DQ8, 1 or more autoantibodies and stage 1 or stage 2 disease. Second, hydroxychloroquine, after its success in rheumatoid arthritis, will be tested in stage 1 individuals. The rationale for this therapeutic, historically used to treat malaria, includes modulation of T cells and interleukins, specifically reductions in Th17 cells in the NOD mouse model of type 1 diabetes. Hydroxychloroquine also has been shown to improve glucose metabolism and insulin sensitivity in type 2 diabetes (49).

In Germany, the Fr1da study, performing general population screening for islet autoantibodies, will also be conducting the Fr1da Insulin Intervention looking at oral insulin in multiple autoantibody-positive subjects enrolled in the natural history study and progression to dysglycemia. This study serves many important purposes, mainly, the feasibility of population screening and seamless enrollment into a prevention study.

Many exciting trials will finish enrollment and follow-up in the next couple of years. As is the challenge with prevention trials, waiting for a clinical endpoint is costly and time-consuming. Large numbers of patients need to be screened and well-powered studies require large numbers of participants, which limit the number of studies able to be performed. Other clinically relevant endpoints are being explored and small, brief studies are being designed to test mechanistic outcomes.

## Conclusion

We have gained incredible knowledge and understanding of the natural history of type 1 diabetes thanks to the international natural history and birth cohort studies described in this review. The presence of increased genetic risk (HLA) and multiple autoantibodies currently provides the most reliable means of predicting type 1 diabetes. However, additional clinical, metabolic, and genetic factors can be assessed to fine-tune that risk. While whole population screening for HLA risk or islet autoimmunity is not yet justified, several groups continue to create networks that will be poised to provide this screening as soon as meaningful prevention is identified. We await the results of several prevention clinical trials and other innovations as we labor to develop a sustainable method of preventing the complex autoimmune process that leads to type 1 diabetes.

As we identify more patients with islet autoimmunity, we must contemplate how to best care for them. All individuals found to have 1 or more islet autoantibodies should, ideally, be referred to contact a type 1 diabetes clinical research center which can be reached through type 1 diabetes supporting agencies, including the JDRF, ADA, NIDDK, and the Type 1 Diabetes TrialNet. Specifically, TrialNet has more than 200 clinical and affiliate centers across the US and worldwide (www.trialnet.org). Through this connection, individuals can decide if enrollment in a prevention clinical trial and/or natural history study is feasible as this is the best way to advance the field of prevention research.

Because of these efforts, a growing number of children are diagnosed with type 1 diabetes prior to the onset of clinical symptoms. There remain no firm guidelines for the follow-up and monitoring of individuals with 1 or more autoantibodies in the general community. Anecdotal evidence is provided and case studies emanating from birth cohorts like TEDDY will help to clarify management options that will undoubtedly vary based on institutional and country-specific preferences. The DPT-1, the Type 1 Diabetes TrialNet international clinical trial collaboration, the SEARCH for Diabetes in Youth Study, Fr1DA, and others will continue to provide valuable information with regards to the potential for population-based screening and the management of patients diagnosed with early type 1 diabetes.

In closing, whether it be through targeted screening of relatives for autoantibodies or population-based screening for high-risk HLA, we must continue to study the natural history of type 1 diabetes and identify patients with beta cell autoimmunity. We feel that primary care providers and subspecialists alike must continue to work together to identify these patients and encourage them to participate in research. Only through these concerted efforts will we move closer to our ultimate goal of preventing and reversing type 1 diabetes.

## Author Contributions

DAS conceptualized this proposal and reviewed/edited the manuscript, LMJ reviewed the current literature and wrote the manuscript, MJH reviewed/edited the manuscript. All authors listed have made a substantial, direct, and intellectual contribution to the work and approved it for publication.

## Conflict of Interest Statement

The authors declare that the research was conducted in the absence of any commercial or financial relationships that could be construed as a potential conflict of interest.

## Abbreviations

HLA, human leukocyte antigen; HbA1c, hemoglobin A1c; GLP-1, glucagon-like peptide-1; NOD, non-obese diabetic.
